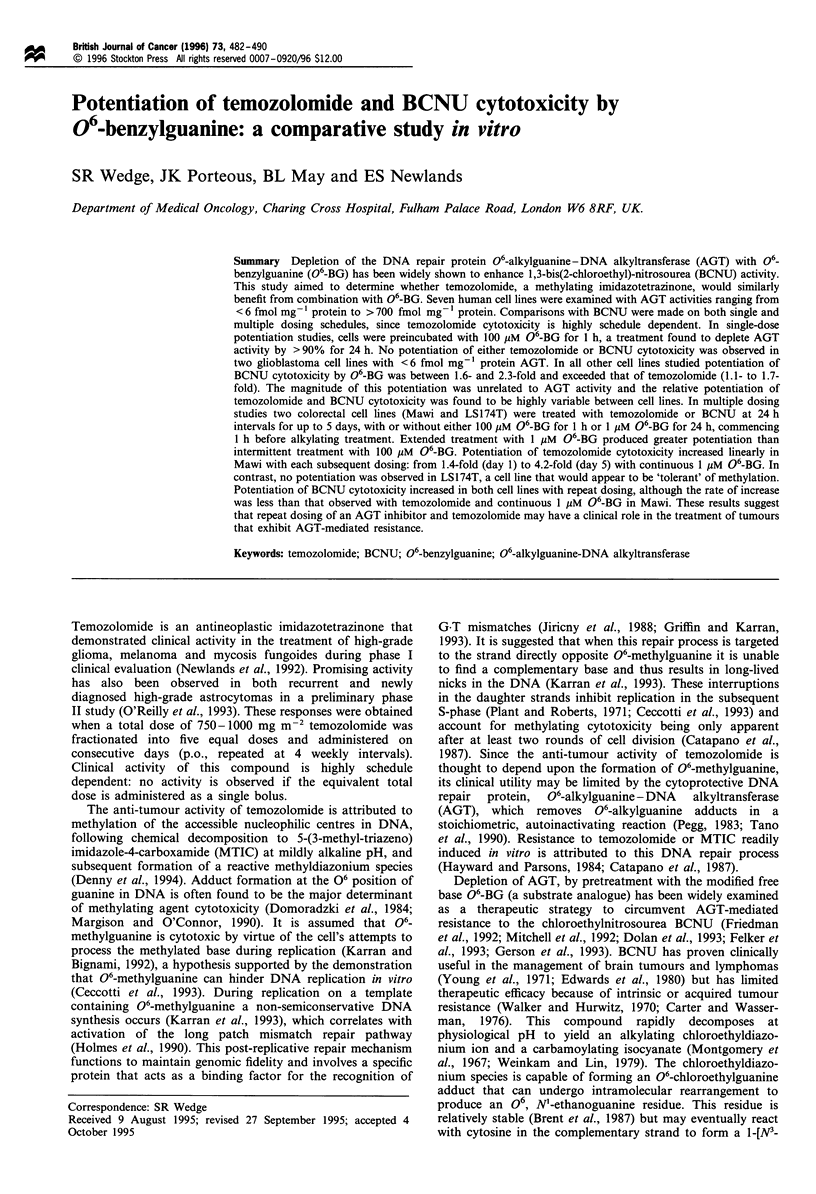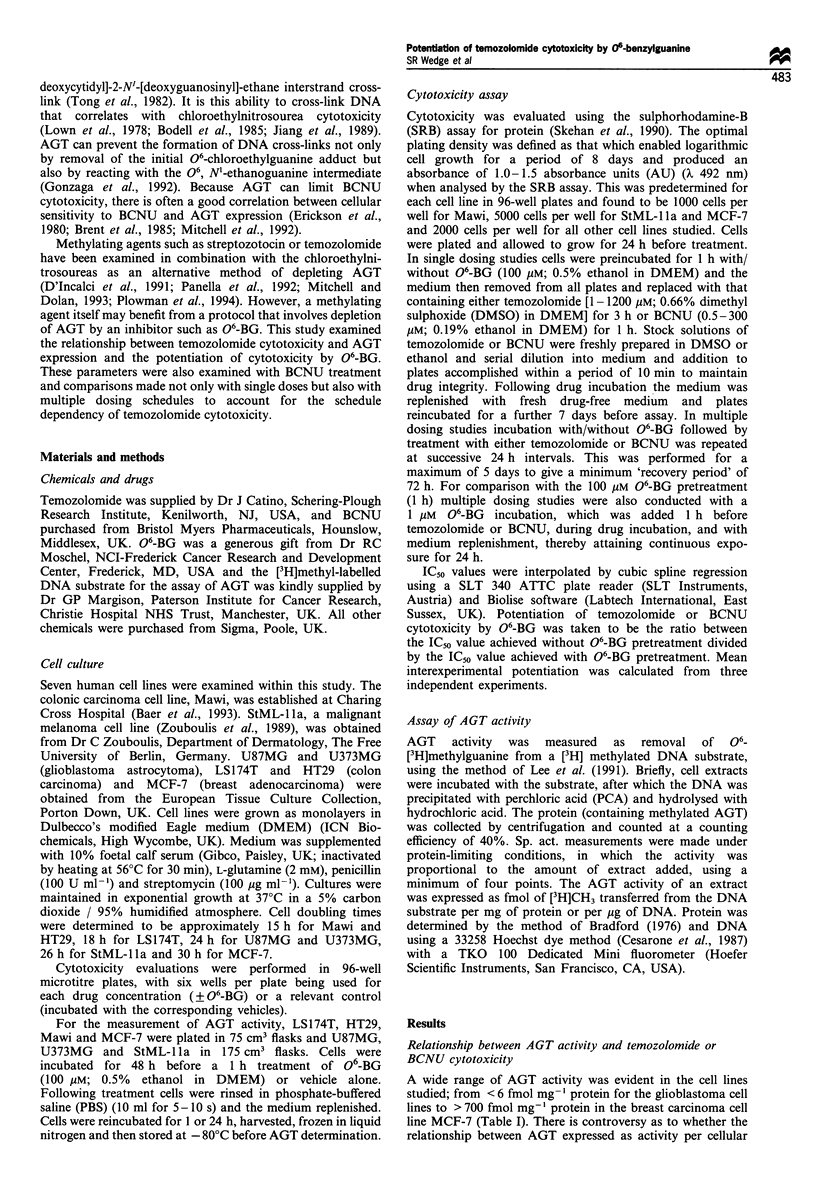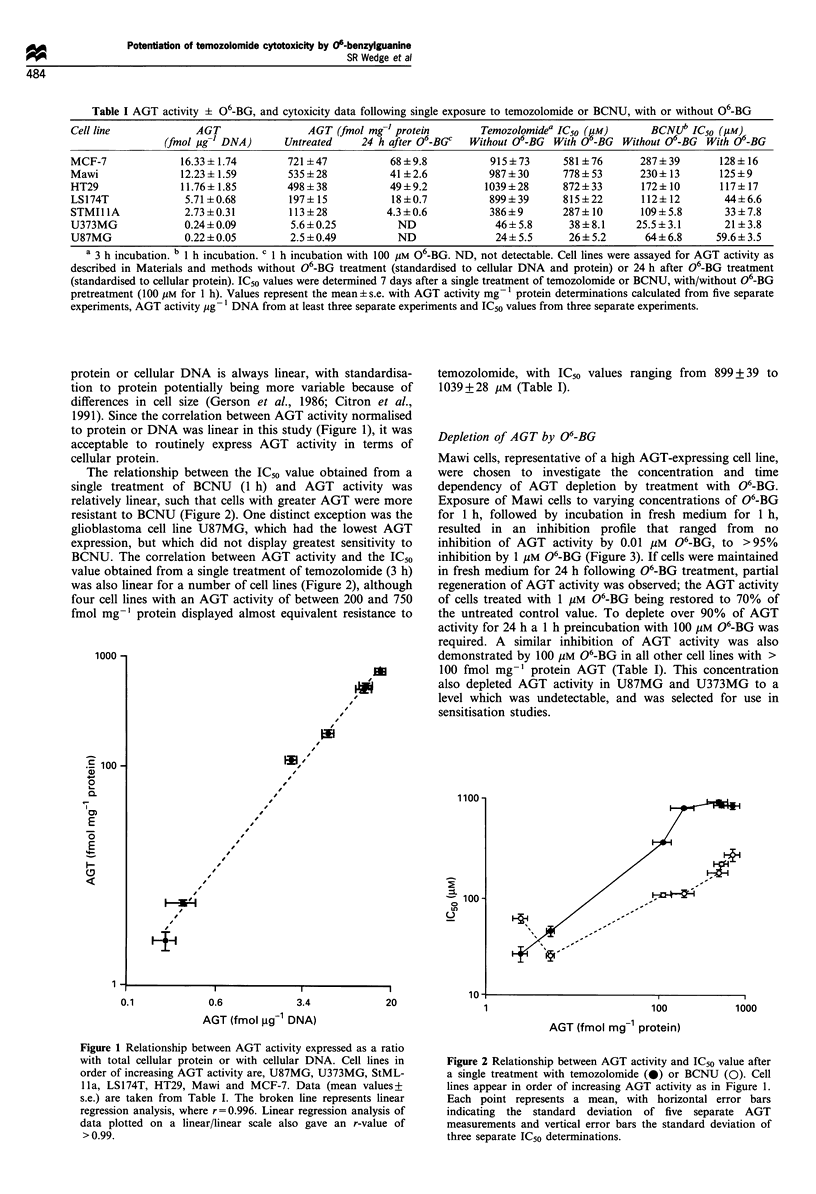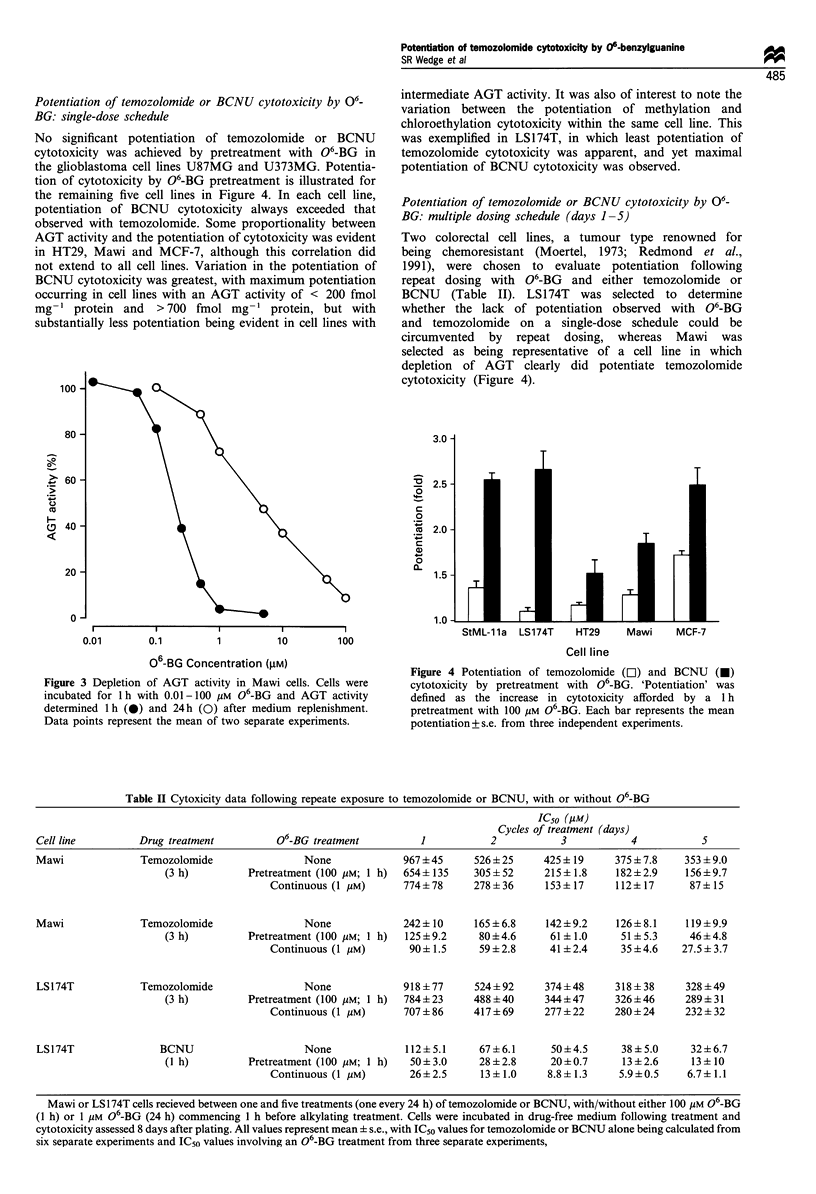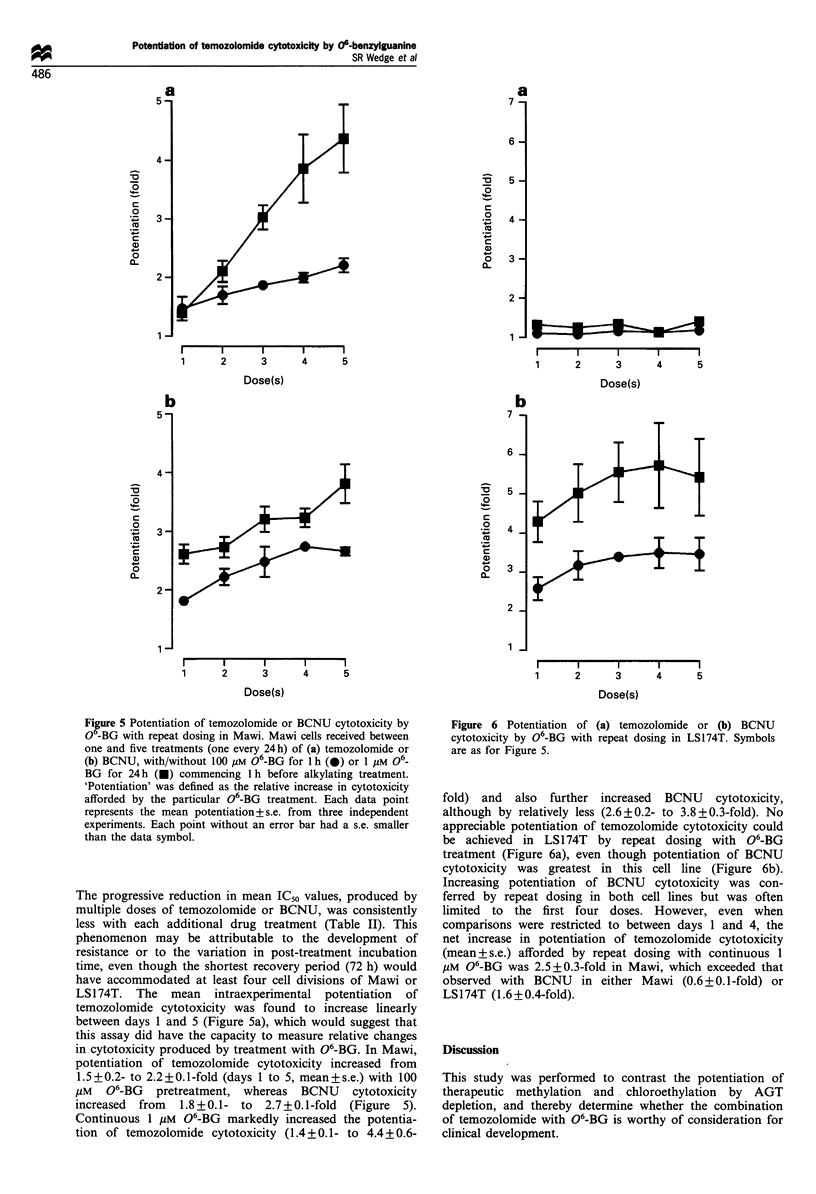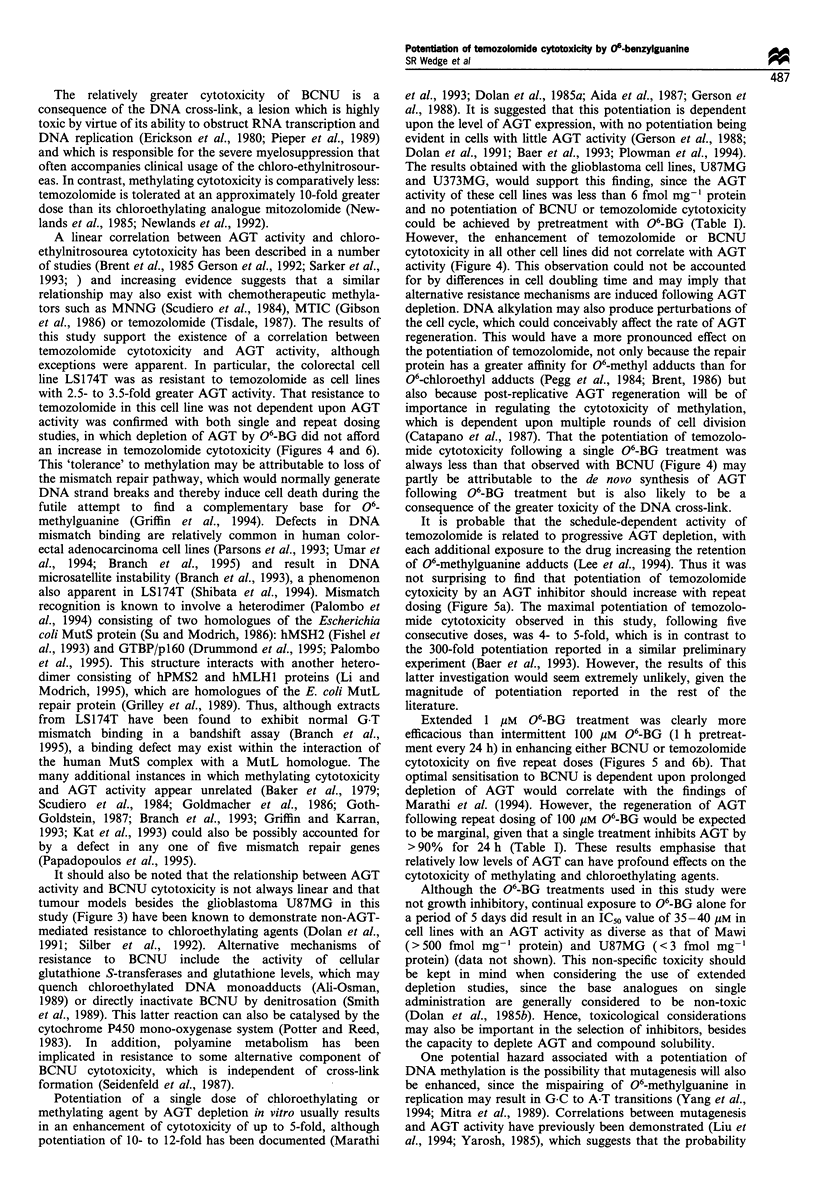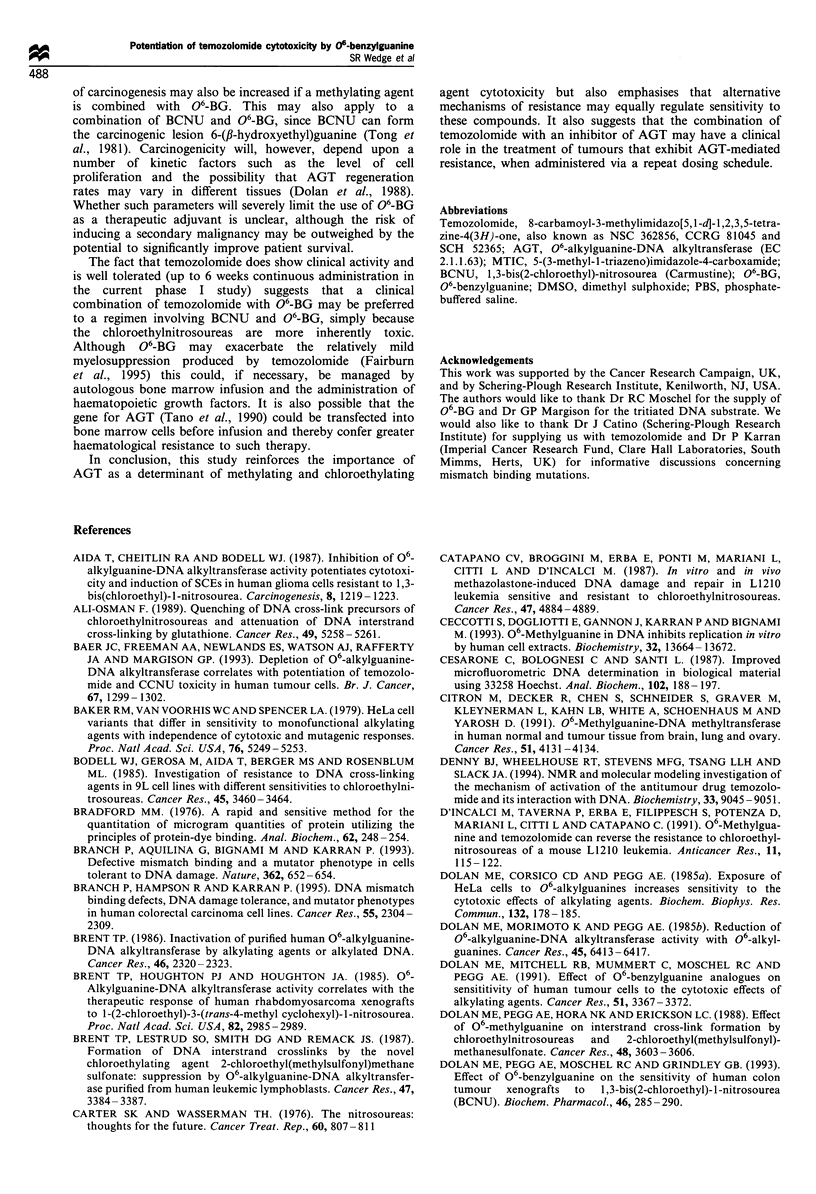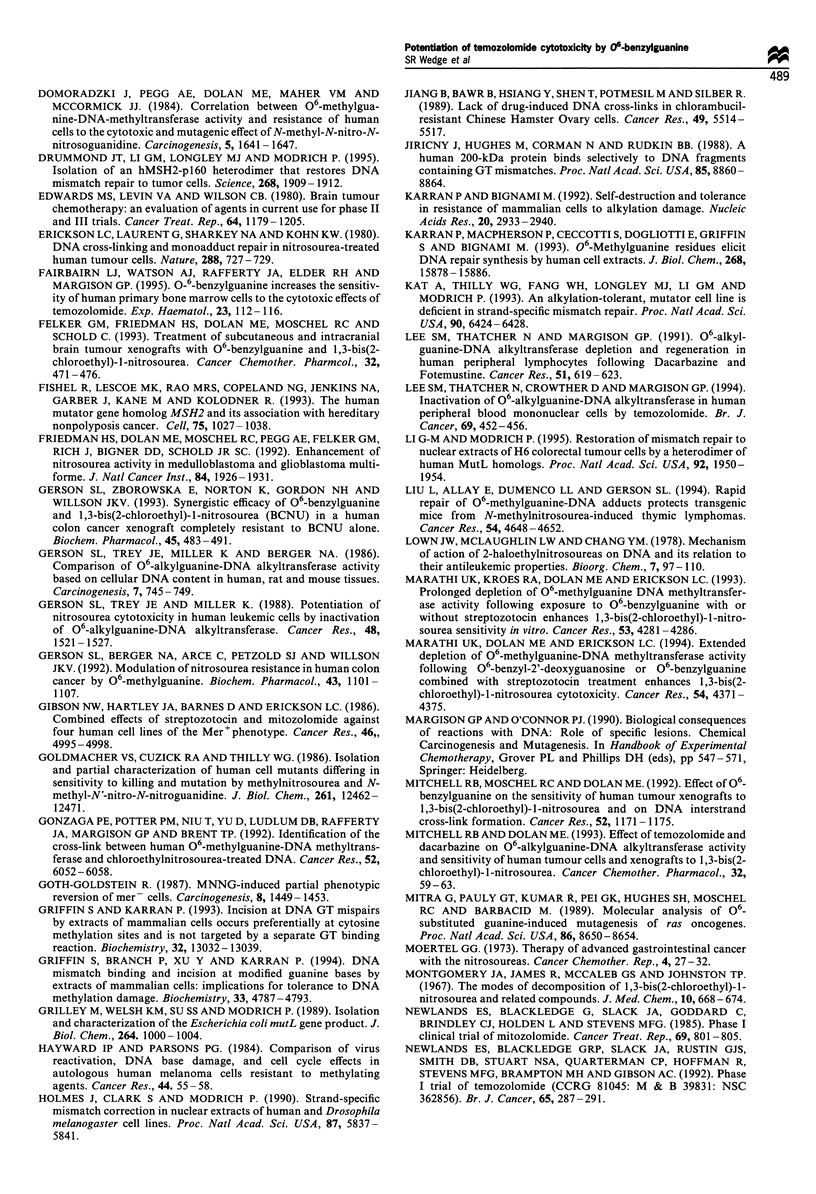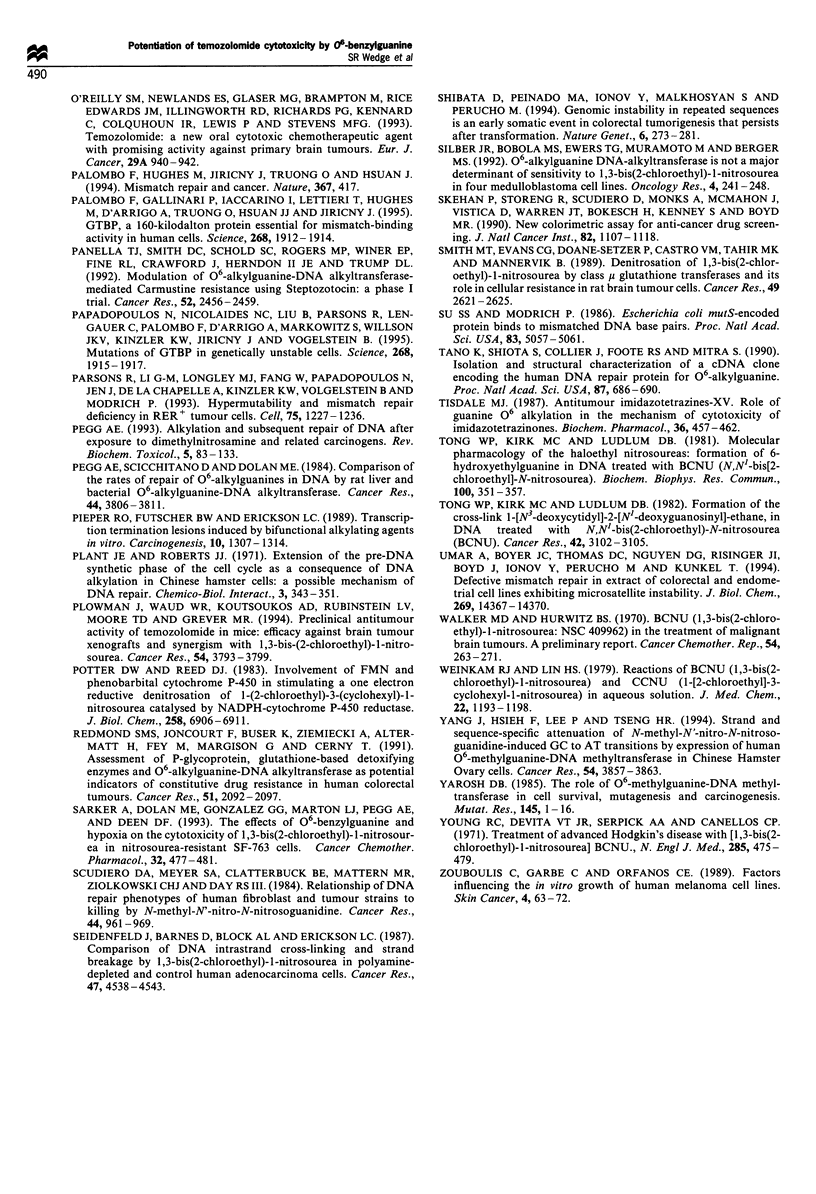# Potentiation of temozolomide and BCNU cytotoxicity by O(6)-benzylguanine: a comparative study in vitro.

**DOI:** 10.1038/bjc.1996.85

**Published:** 1996-02

**Authors:** S. R. Wedge, J. K. Porteus, B. L. May, E. S. Newlands

**Affiliations:** Department of Medical Oncology, Charing Cross Hospital, London, UK.

## Abstract

Depletion of the DNA repair protein O(6)-alkylguanine-DNA alkyltransferase (AGT) with O(6)-benzylguanine (O(6)-BG) has been widely shown to enhance 1,3-bis(2-chloroethyl)-nitrosourea (BCNU) activity. This study aimed to determine whether temozolomide, a methylating imidazotetrazinone, would similarly benefit from combination with O(6)-BG. Seven human cell lines were examined with AGT activities ranging from <6 fmol mg-1 protein to >700 fmol mg-1 protein. Comparisons with BCNU were made on both single and multiple dosing schedules, since temozolomide cytotoxicity is highly schedule dependent. In single-dose potentiation studies, cells were preincubated with 100 microM O(6)-BG for 1 h, a treatment found to deplete AGT activity by >90% for 24 h. No potentiation of either temozolomide or BCNU cytotoxicity was observed in two glioblastoma cell lines with <6 fmol mg-1 protein AGT. In all other cell lines studied potentiation of BCNU toxicity by O(6)-BG was between 1.6- and 2.3-fold and exceeded that of temozolomide (1.1- to 1.7-fold). The magnitude of this potentiation was unrelated to AGT activity and the relative potentiation of temozolomide and BCNU cytotoxicity was found to be highly variable between cell lines. In multiple dosing studies two colorectal cell lines (Mawi and LS174T) were treated with temozolomide or BCNU at 24 h intervals for up to 5 days, with or without either 100 microM O(6)-BG for 1 h or 1 microM O(6)-BG for 24 h, commencing 1 h before alkylating treatment. Extended treatment with 1 microM O(6)-BG produced greater potentiation than intermittent treatment with 100 microM O(6)-BG. Potentiation of temozolomide cytotoxicity increased linearly in Mawi with each subsequent dosing: from 1.4-fold (day 1) to 4.2-fold (day 5) with continuous 1 microM O(6)-BG. In contrast, no potentiation was observed in LS174T, a cell line that would appear to be 'tolerant' of methylation. Potentiation of BNCU cytotoxicity increased in both cell lines with repeat dosing, although the rate of increase was less than that observed with temozolomide and continuous 1 microM O(6)-BG in Mawi. These results suggest that repeat dosing of an AGT inhibitor and temozolomide may have a clinical role in the treatment of tumours that exhibit AGT-mediated resistance.


British Journal of Cancer (1996) 73, 482-490
© 1996 Stockton Press All rights reserved 0007-0920/96 $12.00

### Potentiation of temozolomide and BCNU cytotoxicity by $O^6$-benzylguanine: a comparative study *in vitro*

SR Wedge, JK Porteous, BL May and ES Newlands

*Department of Medical Oncology, Charing Cross Hospital, Fulham Palace Road, London W6 8RF, UK.*

**Summary** Depletion of the DNA repair protein $O^6$-alkylguanine-DNA alkyltransferase (AGT) with $O^6$-benzylguanine ($O^6$-BG) has been widely shown to enhance 1,3-bis(2-chloroethyl)-nitrosourea (BCNU) activity. This study aimed to determine whether temozolomide, a methylating imidazotetrazinone, would similarly benefit from combination with $O^6$-BG. Seven human cell lines were examined with AGT activities ranging from $<6$ fmol $mg^{-1}$ protein to $>700$ fmol $mg^{-1}$ protein. Comparisons with BCNU were made on both single and multiple dosing schedules, since temozolomide cytotoxicity is highly schedule dependent. In single-dose potentiation studies, cells were preincubated with 100 $\mu$M $O^6$-BG for 1 h, a treatment found to deplete AGT activity by $>90\%$ for 24 h. No potentiation of either temozolomide or BCNU cytotoxicity was observed in two glioblastoma cell lines with $<6$ fmol $mg^{-1}$ protein AGT. In all other cell lines studied potentiation of BCNU cytotoxicity by $O^6$-BG was between 1.6- and 2.3-fold and exceeded that of temozolomide (1.1- to 1.7-fold). The magnitude of this potentiation was unrelated to AGT activity and the relative potentiation of temozolomide and BCNU cytotoxicity was found to be highly variable between cell lines. In multiple dosing studies two colorectal cell lines (Mawi and LS174T) were treated with temozolomide or BCNU at 24 h intervals for up to 5 days, with or without either 100 $\mu$M $O^6$-BG for 1 h or 1 $\mu$M $O^6$-BG for 24 h, commencing 1 h before alkylating treatment. Extended treatment with 1 $\mu$M $O^6$-BG produced greater potentiation than intermittent treatment with 100 $\mu$M $O^6$-BG. Potentiation of temozolomide cytotoxicity increased linearly in Mawi with each subsequent dosing: from 1.4-fold (day 1) to 4.2-fold (day 5) with continuous 1 $\mu$M $O^6$-BG. In contrast, no potentiation was observed in LS174T, a cell line that would appear to be 'tolerant' of methylation. Potentiation of BCNU cytotoxicity increased in both cell lines with repeat dosing, although the rate of increase was less than that observed with temozolomide and continuous 1 $\mu$M $O^6$-BG in Mawi. These results suggest that repeat dosing of an AGT inhibitor and temozolomide may have a clinical role in the treatment of tumours that exhibit AGT-mediated resistance.

**Keywords:** temozolomide; BCNU; $O^6$-benzylguanine; $O^6$-alkylguanine-DNA alkyltransferase

Temozolomide is an antineoplastic imidazotetrazinone that demonstrated clinical activity in the treatment of high-grade glioma, melanoma and mycosis fungoides during phase I clinical evaluation (Newlands *et al.*, 1992). Promising activity has also been observed in both recurrent and newly diagnosed high-grade astrocytomas in a preliminary phase II study (O'Reilly *et al.*, 1993). These responses were obtained when a total dose of 750-1000 mg $m^{-2}$ temozolomide was fractionated into five equal doses and administered on consecutive days (p.o., repeated at 4 weekly intervals). Clinical activity of this compound is highly schedule dependent: no activity is observed if the equivalent total dose is administered as a single bolus.

The anti-tumour activity of temozolomide is attributed to methylation of the accessible nucleophilic centres in DNA, following chemical decomposition to 5-(3-methyl-triazeno) imidazole-4-carboxamide (MTIC) at mildly alkaline pH, and subsequent formation of a reactive methyldiazonium species (Denny *et al.*, 1994). Adduct formation at the $O^6$ position of guanine in DNA is often found to be the major determinant of methylating agent cytotoxicity (Domoradzki *et al.*, 1984; Margison and O'Connor, 1990). It is assumed that $O^6$-methylguanine is cytotoxic by virtue of the cell's attempts to process the methylated base during replication (Karran and Bignami, 1992), a hypothesis supported by the demonstration that $O^6$-methylguanine can hinder DNA replication *in vitro* (Ceccotti *et al.*, 1993). During replication on a template containing $O^6$-methylguanine a non-semiconservative DNA synthesis occurs (Karran *et al.*, 1993), which correlates with activation of the long patch mismatch repair pathway (Holmes *et al.*, 1990). This post-replicative repair mechanism functions to maintain genomic fidelity and involves a specific protein that acts as a binding factor for the recognition of G·T mismatches (Jiricny *et al.*, 1988; Griffin and Karran, 1993). It is suggested that when this repair process is targeted to the strand directly opposite $O^6$-methylguanine it is unable to find a complementary base and thus results in long-lived nicks in the DNA (Karran *et al.*, 1993). These interruptions in the daughter strands inhibit replication in the subsequent S-phase (Plant and Roberts, 1971; Ceccotti *et al.*, 1993) and account for methylating cytotoxicity being only apparent after at least two rounds of cell division (Catapano *et al.*, 1987). Since the anti-tumour activity of temozolomide is thought to depend upon the formation of $O^6$-methylguanine, its clinical utility may be limited by the cytoprotective DNA repair protein, $O^6$-alkylguanine-DNA alkyltransferase (AGT), which removes $O^6$-alkylguanine adducts in a stoichiometric, autoinactivating reaction (Pegg, 1983; Tano *et al.*, 1990). Resistance to temozolomide or MTIC readily induced *in vitro* is attributed to this DNA repair process (Hayward and Parsons, 1984; Catapano *et al.*, 1987).

Depletion of AGT, by pretreatment with the modified free base $O^6$-BG (a substrate analogue) has been widely examined as a therapeutic strategy to circumvent AGT-mediated resistance to the chloroethylnitrosourea BCNU (Friedman *et al.*, 1992; Mitchell *et al.*, 1992; Dolan *et al.*, 1993; Felker *et al.*, 1993; Gerson *et al.*, 1993). BCNU has proven clinically useful in the management of brain tumours and lymphomas (Young *et al.*, 1971; Edwards *et al.*, 1980) but has limited therapeutic efficacy because of intrinsic or acquired tumour resistance (Walker and Hurwitz, 1970; Carter and Wasserman, 1976). This compound rapidly decomposes at physiological pH to yield an alkylating chloroethyldiazonium ion and a carbamoylating isocyanate (Montgomery *et al.*, 1967; Weinkam and Lin, 1979). The chloroethyldiazonium species is capable of forming an $O^6$-chloroethylguanine adduct that can undergo intramolecular rearrangement to produce an $O^6$, $N^1$-ethanoguanine residue. This residue is relatively stable (Brent *et al.*, 1987) but may eventually react with cytosine in the complementary strand to form a 1-[$N^3$-

Correspondence: SR Wedge
Received 9 August 1995; revised 27 September 1995; accepted 4 October 1995

deoxycytidyl]-2-$N^1$-[deoxyguanosinyl]-ethane interstrand cross-link (Tong *et al.*, 1982). It is this ability to cross-link DNA that correlates with chloroethylnitrosourea cytotoxicity (Lown *et al.*, 1978; Bodell *et al.*, 1985; Jiang *et al.*, 1989). AGT can prevent the formation of DNA cross-links not only by removal of the initial $O^6$-chloroethylguanine adduct but also by reacting with the $O^6$, $N^1$-ethanoguanine intermediate (Gonzaga *et al.*, 1992). Because AGT can limit BCNU cytotoxicity, there is often a good correlation between cellular sensitivity to BCNU and AGT expression (Erickson *et al.*, 1980; Brent *et al.*, 1985; Mitchell *et al.*, 1992).

Methylating agents such as streptozotocin or temozolomide have been examined in combination with the chloroethylnitrosoureas as an alternative method of depleting AGT (D'Incalci *et al.*, 1991; Panella *et al.*, 1992; Mitchell and Dolan, 1993; Plowman *et al.*, 1994). However, a methylating agent itself may benefit from a protocol that involves depletion of AGT by an inhibitor such as $O^6$-BG. This study examined the relationship between temozolomide cytotoxicity and AGT expression and the potentiation of cytotoxicity by $O^6$-BG. These parameters were also examined with BCNU treatment and comparisons made not only with single doses but also with multiple dosing schedules to account for the schedule dependency of temozolomide cytotoxicity.

### Materials and methods

### *Chemicals and drugs*

Temozolomide was supplied by Dr J Catino, Schering-Plough Research Institute, Kenilworth, NJ, USA, and BCNU purchased from Bristol Myers Pharmaceuticals, Hounslow, Middlesex, UK. $O^6$-BG was a generous gift from Dr RC Moschel, NCI-Frederick Cancer Research and Development Center, Frederick, MD, USA and the [$^3$H]methyl-labelled DNA substrate for the assay of AGT was kindly supplied by Dr GP Margison, Paterson Institute for Cancer Research, Christie Hospital NHS Trust, Manchester, UK. All other chemicals were purchased from Sigma, Poole, UK.

### *Cell culture*

Seven human cell lines were examined within this study. The colonic carcinoma cell line, Mawi, was established at Charing Cross Hospital (Baer *et al.*, 1993). StML-11a, a malignant melanoma cell line (Zouboulis *et al.*, 1989), was obtained from Dr C Zouboulis, Department of Dermatology, The Free University of Berlin, Germany. U87MG and U373MG (glioblastoma astrocytoma), LS174T and HT29 (colon carcinoma) and MCF-7 (breast adenocarcinoma) were obtained from the European Tissue Culture Collection, Porton Down, UK. Cell lines were grown as monolayers in Dulbecco's modified Eagle medium (DMEM) (ICN Biochemicals, High Wycombe, UK). Medium was supplemented with 10% foetal calf serum (Gibco, Paisley, UK; inactivated by heating at 56°C for 30 min), L-glutamine (2 mM), penicillin (100 U $ml^{-1}$) and streptomycin (100 $\mu g$ $ml^{-1}$). Cultures were maintained in exponential growth at 37°C in a 5% carbon dioxide / 95% humidified atmosphere. Cell doubling times were determined to be approximately 15 h for Mawi and HT29, 18 h for LS174T, 24 h for U87MG and U373MG, 26 h for StML-11a and 30 h for MCF-7.

Cytotoxicity evaluations were performed in 96-well microtitre plates, with six wells per plate being used for each drug concentration ($\pm O^6$-BG) or a relevant control (incubated with the corresponding vehicles).

For the measurement of AGT activity, LS174T, HT29, Mawi and MCF-7 were plated in 75 $cm^3$ flasks and U87MG, U373MG and StML-11a in 175 $cm^3$ flasks. Cells were incubated for 48 h before a 1 h treatment of $O^6$-BG (100 $\mu$M; 0.5% ethanol in DMEM) or vehicle alone. Following treatment cells were rinsed in phosphate-buffered saline (PBS) (10 ml for 5–10 s) and the medium replenished. Cells were reincubated for 1 or 24 h, harvested, frozen in liquid nitrogen and then stored at −80°C before AGT determination.

### *Cytotoxicity assay*

Cytotoxicity was evaluated using the sulphorhodamine-B (SRB) assay for protein (Skehan *et al.*, 1990). The optimal plating density was defined as that which enabled logarithmic cell growth for a period of 8 days and produced an absorbance of 1.0–1.5 absorbance units (AU) (λ 492 nm) when analysed by the SRB assay. This was predetermined for each cell line in 96-well plates and found to be 1000 cells per well for Mawi, 5000 cells per well for StML-11a and MCF-7 and 2000 cells per well for all other cell lines studied. Cells were plated and allowed to grow for 24 h before treatment. In single dosing studies cells were preincubated for 1 h with/without $O^6$-BG (100 $\mu$M; 0.5% ethanol in DMEM) and the medium then removed from all plates and replaced with that containing either temozolomide [1–1200 $\mu$M; 0.66% dimethyl sulphoxide (DMSO) in DMEM] for 3 h or BCNU (0.5–300 $\mu$M; 0.19% ethanol in DMEM) for 1 h. Stock solutions of temozolomide or BCNU were freshly prepared in DMSO or ethanol and serial dilution into medium and addition to plates accomplished within a period of 10 min to maintain drug integrity. Following drug incubation the medium was replenished with fresh drug-free medium and plates reincubated for a further 7 days before assay. In multiple dosing studies incubation with/without $O^6$-BG followed by treatment with either temozolomide or BCNU was repeated at successive 24 h intervals. This was performed for a maximum of 5 days to give a minimum 'recovery period' of 72 h. For comparison with the 100 $\mu$M $O^6$-BG pretreatment (1 h) multiple dosing studies were also conducted with a 1 $\mu$M $O^6$-BG incubation, which was added 1 h before temozolomide or BCNU, during drug incubation, and with medium replenishment, thereby attaining continuous exposure for 24 h.

$IC_{50}$ values were interpolated by cubic spline regression using a SLT 340 ATTC plate reader (SLT Instruments, Austria) and Biolise software (Labtech International, East Sussex, UK). Potentiation of temozolomide or BCNU cytotoxicity by $O^6$-BG was taken to be the ratio between the $IC_{50}$ value achieved without $O^6$-BG pretreatment divided by the $IC_{50}$ value achieved with $O^6$-BG pretreatment. Mean interexperimental potentiation was calculated from three independent experiments.

### *Assay of AGT activity*

AGT activity was measured as removal of $O^6$-[$^3$H]methylguanine from a [$^3$H] methylated DNA substrate, using the method of Lee *et al.* (1991). Briefly, cell extracts were incubated with the substrate, after which the DNA was precipitated with perchloric acid (PCA) and hydrolysed with hydrochloric acid. The protein (containing methylated AGT) was collected by centrifugation and counted at a counting efficiency of 40%. Sp. act. measurements were made under protein-limiting conditions, in which the activity was proportional to the amount of extract added, using a minimum of four points. The AGT activity of an extract was expressed as fmol of [$^3$H]$CH_3$ transferred from the DNA substrate per mg of protein or per $\mu g$ of DNA. Protein was determined by the method of Bradford (1976) and DNA using a 33258 Hoechst dye method (Cesarone *et al.*, 1987) with a TKO 100 Dedicated Mini fluorometer (Hoefer Scientific Instruments, San Francisco, CA, USA).

### Results

### *Relationship between AGT activity and temozolomide or BCNU cytotoxicity*

A wide range of AGT activity was evident in the cell lines studied; from <6 fmol $mg^{-1}$ protein for the glioblastoma cell lines to >700 fmol $mg^{-1}$ protein in the breast carcinoma cell line MCF-7 (Table I). There is controversy as to whether the relationship between AGT expressed as activity per cellular

**Table I** AGT activity ± $O^6$-BG, and cytoxicity data following single exposure to temozolomide or BCNU, with or without $O^6$-BG

| *Cell line* | *AGT (fmol $\mu g^{-1}$ DNA)* | *AGT (fmol $mg^{-1}$ protein* Untreated | 24 h after $O^6$-BG[c] | *Temozolomide[a] $IC_{50}$ (μM)* Without $O^6$-BG | With $O^6$-BG | *BCNU[b] $IC_{50}$ (μM)* Without $O^6$-BG | With $O^6$-BG |
|---|---|---|---|---|---|---|---|
| MCF-7 | 16.33 ± 1.74 | 721 ± 47 | 68 ± 9.8 | 915 ± 73 | 581 ± 76 | 287 ± 39 | 128 ± 16 |
| Mawi | 12.23 ± 1.59 | 535 ± 28 | 41 ± 2.6 | 987 ± 30 | 778 ± 53 | 230 ± 13 | 125 ± 9 |
| HT29 | 11.76 ± 1.85 | 498 ± 38 | 49 ± 9.2 | 1039 ± 28 | 872 ± 33 | 172 ± 10 | 117 ± 17 |
| LS174T | 5.71 ± 0.68 | 197 ± 15 | 18 ± 0.7 | 899 ± 39 | 815 ± 22 | 112 ± 12 | 44 ± 6.6 |
| STMI11A | 2.73 ± 0.31 | 113 ± 28 | 4.3 ± 0.6 | 386 ± 9 | 287 ± 10 | 109 ± 5.8 | 33 ± 7.8 |
| U373MG | 0.24 ± 0.09 | 5.6 ± 0.25 | ND | 46 ± 5.8 | 38 ± 8.1 | 25.5 ± 3.1 | 21 ± 3.8 |
| U87MG | 0.22 ± 0.05 | 2.5 ± 0.49 | ND | 24 ± 5.5 | 26 ± 5.2 | 64 ± 6.8 | 59.6 ± 3.5 |

[a] 3 h incubation. [b] 1 h incubation. [c] 1 h incubation with 100 μM $O^6$-BG. ND, not detectable. Cell lines were assayed for AGT activity as described in Materials and methods without $O^6$-BG treatment (standardised to cellular DNA and protein) or 24 h after $O^6$-BG treatment (standardised to cellular protein). $IC_{50}$ values were determined 7 days after a single treatment of temozolomide or BCNU, with/without $O^6$-BG pretreatment (100 μM for 1 h). Values represent the mean ± s.e. with AGT activity $mg^{-1}$ protein determinations calculated from five separate experiments, AGT activity $\mu g^{-1}$ DNA from at least three separate experiments and $IC_{50}$ values from three separate experiments.

protein or cellular DNA is always linear, with standardisation to protein potentially being more variable because of differences in cell size (Gerson *et al.*, 1986; Citron *et al.*, 1991). Since the correlation between AGT activity normalised to protein or DNA was linear in this study (Figure 1), it was acceptable to routinely express AGT activity in terms of cellular protein.

The relationship between the $IC_{50}$ value obtained from a single treatment of BCNU (1 h) and AGT activity was relatively linear, such that cells with greater AGT were more resistant to BCNU (Figure 2). One distinct exception was the glioblastoma cell line U87MG, which had the lowest AGT expression, but which did not display greatest sensitivity to BCNU. The correlation between AGT activity and the $IC_{50}$ value obtained from a single treatment of temozolomide (3 h) was also linear for a number of cell lines (Figure 2), although four cell lines with an AGT activity of between 200 and 750 fmol $mg^{-1}$ protein displayed almost equivalent resistance to temozolomide, with $IC_{50}$ values ranging from 899 ± 39 to 1039 ± 28 μM (Table I).

**Figure 1** Relationship between AGT activity expressed as a ratio with total cellular protein or with cellular DNA. Cell lines in order of increasing AGT activity are, U87MG, U373MG, StML-11a, LS174T, HT29, Mawi and MCF-7. Data (mean values ± s.e.) are taken from Table I. The broken line represents linear regression analysis, where $r = 0.996$. Linear regression analysis of data plotted on a linear/linear scale also gave an $r$-value of $> 0.99$.

### *Depletion of AGT by $O^6$-BG*

Mawi cells, representative of a high AGT-expressing cell line, were chosen to investigate the concentration and time dependency of AGT depletion by treatment with $O^6$-BG. Exposure of Mawi cells to varying concentrations of $O^6$-BG for 1 h, followed by incubation in fresh medium for 1 h, resulted in an inhibition profile that ranged from no inhibition of AGT activity by 0.01 μM $O^6$-BG, to >95% inhibition by 1 μM $O^6$-BG (Figure 3). If cells were maintained in fresh medium for 24 h following $O^6$-BG treatment, partial regeneration of AGT activity was observed; the AGT activity of cells treated with 1 μM $O^6$-BG being restored to 70% of the untreated control value. To deplete over 90% of AGT activity for 24 h a 1 h preincubation with 100 μM $O^6$-BG was required. A similar inhibition of AGT activity was also demonstrated by 100 μM $O^6$-BG in all other cell lines with > 100 fmol $mg^{-1}$ protein AGT (Table I). This concentration also depleted AGT activity in U87MG and U373MG to a level which was undetectable, and was selected for use in sensitisation studies.

**Figure 2** Relationship between AGT activity and $IC_{50}$ value after a single treatment with temozolomide (●) or BCNU (○). Cell lines appear in order of increasing AGT activity as in Figure 1. Each point represents a mean, with horizontal error bars indicating the standard deviation of five separate AGT measurements and vertical error bars the standard deviation of three separate $IC_{50}$ determinations.

*Potentiation of temozolomide or BCNU cytotoxicity by $O^6$-BG: single-dose schedule*

No significant potentiation of temozolomide or BCNU cytotoxicity was achieved by pretreatment with $O^6$-BG in the glioblastoma cell lines U87MG and U373MG. Potentiation of cytotoxicity by $O^6$-BG pretreatment is illustrated for the remaining five cell lines in Figure 4. In each cell line, potentiation of BCNU cytotoxicity always exceeded that observed with temozolomide. Some proportionality between AGT activity and the potentiation of cytotoxicity was evident in HT29, Mawi and MCF-7, although this correlation did not extend to all cell lines. Variation in the potentiation of BCNU cytotoxicity was greatest, with maximum potentiation occurring in cell lines with an AGT activity of < 200 fmol $mg^{-1}$ protein and >700 fmol $mg^{-1}$ protein, but with substantially less potentiation being evident in cell lines with intermediate AGT activity. It was also of interest to note the variation between the potentiation of methylation and chloroethylation cytotoxicity within the same cell line. This was exemplified in LS174T, in which least potentiation of temozolomide cytotoxicity was apparent, and yet maximal potentiation of BCNU cytotoxicity was observed.

**Figure 3** Depletion of AGT activity in Mawi cells. Cells were incubated for 1 h with 0.01–100 μM $O^6$-BG and AGT activity determined 1 h (●) and 24 h (○) after medium replenishment. Data points represent the mean of two separate experiments.

*Potentiation of temozolomide or BCNU cytotoxicity by $O^6$-BG: multiple dosing schedule (days 1–5)*

Two colorectal cell lines, a tumour type renowned for being chemoresistant (Moertel, 1973; Redmond et al., 1991), were chosen to evaluate potentiation following repeat dosing with $O^6$-BG and either temozolomide or BCNU (Table II). LS174T was selected to determine whether the lack of potentiation observed with $O^6$-BG and temozolomide on a single-dose schedule could be circumvented by repeat dosing, whereas Mawi was selected as being representative of a cell line in which depletion of AGT clearly did potentiate temozolomide cytotoxicity (Figure 4).

**Figure 4** Potentiation of temozolomide (□) and BCNU (■) cytotoxicity by pretreatment with $O^6$-BG. 'Potentiation' was defined as the increase in cytotoxicity afforded by a 1 h pretreatment with 100 μM $O^6$-BG. Each bar represents the mean potentiation ± s.e. from three independent experiments.

**Table II** Cytoxicity data following repeate exposure to temozolomide or BCNU, with or without $O^6$-BG

| | | | $IC_{50}$ (μM) Cycles of treatment (days) | | | | |
|---|---|---|---|---|---|---|---|
| *Cell line* | *Drug treatment* | *$O^6$-BG treatment* | *1* | *2* | *3* | *4* | *5* |
| Mawi | Temozolomide | None | 967±45 | 526±25 | 425±19 | 375±7.8 | 353±9.0 |
| | (3 h) | Pretreatment (100 μM; 1 h) | 654±135 | 305±52 | 215±1.8 | 182±2.9 | 156±9.7 |
| | | Continuous (1 μM) | 774±78 | 278±36 | 153±17 | 112±17 | 87±15 |
| Mawi | Temozolomide | None | 242±10 | 165±6.8 | 142±9.2 | 126±8.1 | 119±9.9 |
| | (3 h) | Pretreatment (100 μM; 1 h) | 125±9.2 | 80±4.6 | 61±1.0 | 51±5.3 | 46±4.8 |
| | | Continuous (1 μM) | 90±1.5 | 59±2.8 | 41±2.4 | 35±4.6 | 27.5±3.7 |
| LS174T | Temozolomide | None | 918±77 | 524±92 | 374±48 | 318±38 | 328±49 |
| | (3 h) | Pretreatment (100 μM; 1 h) | 784±23 | 488±40 | 344±47 | 326±46 | 289±31 |
| | | Continuous (1 μM) | 707±86 | 417±69 | 277±22 | 280±24 | 232±32 |
| LS174T | BCNU | None | 112±5.1 | 67±6.1 | 50±4.5 | 38±5.0 | 32±6.7 |
| | (1 h) | Pretreatment (100 μM; 1 h) | 50±3.0 | 28±2.8 | 20±0.7 | 13±2.6 | 13±10 |
| | | Continuous (1 μM) | 26±2.5 | 13±1.0 | 8.8±1.3 | 5.9±0.5 | 6.7±1.1 |

Mawi or LS174T cells recieved between one and five treatments (one every 24 h) of temozolomide or BCNU, with/without either 100 μM $O^6$-BG (1 h) or 1 μM $O^6$-BG (24 h) commencing 1 h before alkylating treatment. Cells were incubated in drug-free medium following treatment and cytotoxicity assessed 8 days after plating. All values represent mean ± s.e., with $IC_{50}$ values for temozolomide or BCNU alone being calculated from six separate experiments and $IC_{50}$ values involving an $O^6$-BG treatment from three separate experiments,

**Figure 5** Potentiation of temozolomide or BCNU cytotoxicity by $O^6$-BG with repeat dosing in Mawi. Mawi cells received between one and five treatments (one every 24 h) of (**a**) temozolomide or (**b**) BCNU, with/without 100 $\mu$M $O^6$-BG for 1 h (●) or 1 $\mu$M $O^6$-BG for 24 h (■) commencing 1 h before alkylating treatment. 'Potentiation' was defined as the relative increase in cytotoxicity afforded by the particular $O^6$-BG treatment. Each data point represents the mean potentiation ± s.e. from three independent experiments. Each point without an error bar had a s.e. smaller than the data symbol.

**Figure 6** Potentiation of (**a**) temozolomide or (**b**) BCNU cytotoxicity by $O^6$-BG with repeat dosing in LS174T. Symbols are as for Figure 5.

The progressive reduction in mean $IC_{50}$ values, produced by multiple doses of temozolomide or BCNU, was consistently less with each additional drug treatment (Table II). This phenomenon may be attributable to the development of resistance or to the variation in post-treatment incubation time, even though the shortest recovery period (72 h) would have accommodated at least four cell divisions of Mawi or LS174T. The mean intraexperimental potentiation of temozolomide cytotoxicity was found to increase linearly between days 1 and 5 (Figure 5a), which would suggest that this assay did have the capacity to measure relative changes in cytotoxicity produced by treatment with $O^6$-BG. In Mawi, potentiation of temozolomide cytotoxicity increased from 1.5 ± 0.2- to 2.2 ± 0.1-fold (days 1 to 5, mean ± s.e.) with 100 $\mu$M $O^6$-BG pretreatment, whereas BCNU cytotoxicity increased from 1.8 ± 0.1- to 2.7 ± 0.1-fold (Figure 5). Continuous 1 $\mu$M $O^6$-BG markedly increased the potentiation of temozolomide cytotoxicity (1.4 ± 0.1- to 4.4 ± 0.6-fold) and also further increased BCNU cytotoxicity, although by relatively less (2.6 ± 0.2- to 3.8 ± 0.3-fold). No appreciable potentiation of temozolomide cytotoxicity could be achieved in LS174T by repeat dosing with $O^6$-BG treatment (Figure 6a), even though potentiation of BCNU cytotoxicity was greatest in this cell line (Figure 6b). Increasing potentiation of BCNU cytotoxicity was conferred by repeat dosing in both cell lines but was often limited to the first four doses. However, even when comparisons were restricted to between days 1 and 4, the net increase in potentiation of temozolomide cytotoxicity (mean ± s.e.) afforded by repeat dosing with continuous 1 $\mu$M $O^6$-BG was 2.5 ± 0.3-fold in Mawi, which exceeded that observed with BCNU in either Mawi (0.6 ± 0.1-fold) or LS174T (1.6 ± 0.4-fold).

### Discussion

This study was performed to contrast the potentiation of therapeutic methylation and chloroethylation by AGT depletion, and thereby determine whether the combination of temozolomide with $O^6$-BG is worthy of consideration for clinical development.

The relatively greater cytotoxicity of BCNU is a consequence of the DNA cross-link, a lesion which is highly toxic by virtue of its ability to obstruct RNA transcription and DNA replication (Erickson *et al.*, 1980; Pieper *et al.*, 1989) and which is responsible for the severe myelosuppression that often accompanies clinical usage of the chloro-ethylnitrosoureas. In contrast, methylating cytotoxicity is comparatively less: temozolomide is tolerated at an approximately 10-fold greater dose than its chloroethylating analogue mitozolomide (Newlands *et al.*, 1985; Newlands *et al.*, 1992).

A linear correlation between AGT activity and chloroethylnitrosourea cytotoxicity has been described in a number of studies (Brent *et al.*, 1985 Gerson *et al.*, 1992; Sarker *et al.*, 1993; ) and increasing evidence suggests that a similar relationship may also exist with chemotherapeutic methylators such as MNNG (Scudiero *et al.*, 1984), MTIC (Gibson *et al.*, 1986) or temozolomide (Tisdale, 1987). The results of this study support the existence of a correlation between temozolomide cytotoxicity and AGT activity, although exceptions were apparent. In particular, the colorectal cell line LS174T was as resistant to temozolomide as cell lines with 2.5- to 3.5-fold greater AGT activity. That resistance to temozolomide in this cell line was not dependent upon AGT activity was confirmed with both single and repeat dosing studies, in which depletion of AGT by $O^6$-BG did not afford an increase in temozolomide cytotoxicity (Figures 4 and 6). This 'tolerance' to methylation may be attributable to loss of the mismatch repair pathway, which would normally generate DNA strand breaks and thereby induce cell death during the futile attempt to find a complementary base for $O^6$-methylguanine (Griffin *et al.*, 1994). Defects in DNA mismatch binding are relatively common in human colorectal adenocarcinoma cell lines (Parsons *et al.*, 1993; Umar *et al.*, 1994; Branch *et al.*, 1995) and result in DNA microsatellite instability (Branch *et al.*, 1993), a phenomenon also apparent in LS174T (Shibata *et al.*, 1994). Mismatch recognition is known to involve a heterodimer (Palombo *et al.*, 1994) consisting of two homologues of the *Escherichia coli* MutS protein (Su and Modrich, 1986): hMSH2 (Fishel *et al.*, 1993) and GTBP/p160 (Drummond *et al.*, 1995; Palombo *et al.*, 1995). This structure interacts with another heterodimer consisting of hPMS2 and hMLH1 proteins (Li and Modrich, 1995), which are homologues of the *E. coli* MutL repair protein (Grilley *et al.*, 1989). Thus, although extracts from LS174T have been found to exhibit normal G·T mismatch binding in a bandshift assay (Branch *et al.*, 1995), a binding defect may exist within the interaction of the human MutS complex with a MutL homologue. The many additional instances in which methylating cytotoxicity and AGT activity appear unrelated (Baker *et al.*, 1979; Scudiero *et al.*, 1984; Goldmacher *et al.*, 1986; Goth-Goldstein, 1987; Branch *et al.*, 1993; Griffin and Karran, 1993; Kat *et al.*, 1993) could also be possibly accounted for by a defect in any one of five mismatch repair genes (Papadopoulos *et al.*, 1995).

It should also be noted that the relationship between AGT activity and BCNU cytotoxicity is not always linear and that tumour models besides the glioblastoma U87MG in this study (Figure 3) have been known to demonstrate non-AGT-mediated resistance to chloroethylating agents (Dolan *et al.*, 1991; Silber *et al.*, 1992). Alternative mechanisms of resistance to BCNU include the activity of cellular glutathione *S*-transferases and glutathione levels, which may quench chloroethylated DNA monoadducts (Ali-Osman, 1989) or directly inactivate BCNU by denitrosation (Smith *et al.*, 1989). This latter reaction can also be catalysed by the cytochrome P450 mono-oxygenase system (Potter and Reed, 1983). In addition, polyamine metabolism has been implicated in resistance to some alternative component of BCNU cytotoxicity, which is independent of cross-link formation (Seidenfeld *et al.*, 1987).

Potentiation of a single dose of chloroethylating or methylating agent by AGT depletion *in vitro* usually results in an enhancement of cytotoxicity of up to 5-fold, although potentiation of 10- to 12-fold has been documented (Marathi *et al.*, 1993; Dolan *et al.*, 1985*a*; Aida *et al.*, 1987; Gerson *et al.*, 1988). It is suggested that this potentiation is dependent upon the level of AGT expression, with no potentiation being evident in cells with little AGT activity (Gerson *et al.*, 1988; Dolan *et al.*, 1991; Baer *et al.*, 1993; Plowman *et al.*, 1994). The results obtained with the glioblastoma cell lines, U87MG and U373MG, would support this finding, since the AGT activity of these cell lines was less than 6 fmol $mg^{-1}$ protein and no potentiation of BCNU or temozolomide cytotoxicity could be achieved by pretreatment with $O^6$-BG (Table I). However, the enhancement of temozolomide or BCNU cytotoxicity in all other cell lines did not correlate with AGT activity (Figure 4). This observation could not be accounted for by differences in cell doubling time and may imply that alternative resistance mechanisms are induced following AGT depletion. DNA alkylation may also produce perturbations of the cell cycle, which could conceivably affect the rate of AGT regeneration. This would have a more pronounced effect on the potentiation of temozolomide, not only because the repair protein has a greater affinity for $O^6$-methyl adducts than for $O^6$-chloroethyl adducts (Pegg *et al.*, 1984; Brent, 1986) but also because post-replicative AGT regeneration will be of importance in regulating the cytotoxicity of methylation, which is dependent upon multiple rounds of cell division (Catapano *et al.*, 1987). That the potentiation of temozolomide cytotoxicity following a single $O^6$-BG treatment was always less than that observed with BCNU (Figure 4) may partly be attributable to the *de novo* synthesis of AGT following $O^6$-BG treatment but is also likely to be a consequence of the greater toxicity of the DNA cross-link.

It is probable that the schedule-dependent activity of temozolomide is related to progressive AGT depletion, with each additional exposure to the drug increasing the retention of $O^6$-methylguanine adducts (Lee *et al.*, 1994). Thus it was not surprising to find that potentiation of temozolomide cytoxicity by an AGT inhibitor should increase with repeat dosing (Figure 5a). The maximal potentiation of temozolomide cytotoxicity observed in this study, following five consecutive doses, was 4- to 5-fold, which is in contrast to the 300-fold potentiation reported in a similar preliminary experiment (Baer *et al.*, 1993). However, the results of this latter investigation would seem extremely unlikely, given the magnitude of potentiation reported in the rest of the literature.

Extended 1 $\mu$M $O^6$-BG treatment was clearly more efficacious than intermittent 100 $\mu$M $O^6$-BG (1 h pretreatment every 24 h) in enhancing either BCNU or temozolomide cytotoxicity on five repeat doses (Figures 5 and 6b). That optimal sensitisation to BCNU is dependent upon prolonged depletion of AGT would correlate with the findings of Marathi *et al.* (1994). However, the regeneration of AGT following repeat dosing of 100 $\mu$M $O^6$-BG would be expected to be marginal, given that a single treatment inhibits AGT by >90% for 24 h (Table I). These results emphasise that relatively low levels of AGT can have profound effects on the cytotoxicity of methylating and chloroethylating agents.

Although the $O^6$-BG treatments used in this study were not growth inhibitory, continual exposure to $O^6$-BG alone for a period of 5 days did result in an $IC_{50}$ value of 35–40 $\mu$M in cell lines with an AGT activity as diverse as that of Mawi (>500 fmol $mg^{-1}$ protein) and U87MG (<3 fmol $mg^{-1}$ protein) (data not shown). This non-specific toxicity should be kept in mind when considering the use of extended depletion studies, since the base analogues on single administration are generally considered to be non-toxic (Dolan *et al.*, 1985*b*). Hence, toxicological considerations may also be important in the selection of inhibitors, besides the capacity to deplete AGT and compound solubility.

One potential hazard associated with a potentiation of DNA methylation is the possibility that mutagenesis will also be enhanced, since the mispairing of $O^6$-methylguanine in replication may result in G·C to A·T transitions (Yang *et al.*, 1994; Mitra *et al.*, 1989). Correlations between mutagenesis and AGT activity have previously been demonstrated (Liu *et al.*, 1994; Yarosh, 1985), which suggests that the probability of carcinogenesis may also be increased if a methylating agent is combined with $O^6$-BG. This may also apply to a combination of BCNU and $O^6$-BG, since BCNU can form the carcinogenic lesion 6-(β-hydroxyethyl)guanine (Tong *et al.*, 1981). Carcinogenicity will, however, depend upon a number of kinetic factors such as the level of cell proliferation and the possibility that AGT regeneration rates may vary in different tissues (Dolan *et al.*, 1988). Whether such parameters will severely limit the use of $O^6$-BG as a therapeutic adjuvant is unclear, although the risk of inducing a secondary malignancy may be outweighed by the potential to significantly improve patient survival.

The fact that temozolomide does show clinical activity and is well tolerated (up to 6 weeks continuous administration in the current phase I study) suggests that a clinical combination of temozolomide with $O^6$-BG may be preferred to a regimen involving BCNU and $O^6$-BG, simply because the chloroethylnitrosoureas are more inherently toxic. Although $O^6$-BG may exacerbate the relatively mild myelosuppression produced by temozolomide (Fairburn *et al.*, 1995) this could, if necessary, be managed by autologous bone marrow infusion and the administration of haematopoietic growth factors. It is also possible that the gene for AGT (Tano *et al.*, 1990) could be transfected into bone marrow cells before infusion and thereby confer greater haematological resistance to such therapy.

In conclusion, this study reinforces the importance of AGT as a determinant of methylating and chloroethylating agent cytotoxicity but also emphasises that alternative mechanisms of resistance may equally regulate sensitivity to these compounds. It also suggests that the combination of temozolomide with an inhibitor of AGT may have a clinical role in the treatment of tumours that exhibit AGT-mediated resistance, when administered via a repeat dosing schedule.

### Abbreviations

Temozolomide, 8-carbamoyl-3-methylimidazo[5,1-*d*]-1,2,3,5-tetrazine-4(3*H*)-one, also known as NSC 362856, CCRG 81045 and SCH 52365; AGT, $O^6$-alkylguanine-DNA alkyltransferase (EC 2.1.1.63); MTIC, 5-(3-methyl-1-triazeno)imidazole-4-carboxamide; BCNU, 1,3-bis(2-chloroethyl)-nitrosourea (Carmustine); $O^6$-BG, $O^6$-benzylguanine; DMSO, dimethyl sulphoxide; PBS, phosphate-buffered saline.

### Acknowledgements

This work was supported by the Cancer Research Campaign, UK, and by Schering-Plough Research Institute, Kenilworth, NJ, USA. The authors would like to thank Dr RC Moschel for the supply of $O^6$-BG and Dr GP Margison for the tritiated DNA substrate. We would also like to thank Dr J Catino (Schering-Plough Research Institute) for supplying us with temozolomide and Dr P Karran (Imperial Cancer Research Fund, Clare Hall Laboratories, South Mimms, Herts, UK) for informative discussions concerning mismatch binding mutations.

### References

AIDA T, CHEITLIN RA AND BODELL WJ. (1987). Inhibition of $O^6$-alkylguanine-DNA alkyltransferase activity potentiates cytotoxicity and induction of SCEs in human glioma cells resistant to 1,3-bis(chloroethyl)-1-nitrosourea. *Carcinogenesis*, **8**, 1219–1223.

ALI-OSMAN F. (1989). Quenching of DNA cross-link precursors of chloroethylnitrosoureas and attenuation of DNA interstrand cross-linking by glutathione. *Cancer Res.*, **49**, 5258–5261.

BAER JC, FREEMAN AA, NEWLANDS ES, WATSON AJ, RAFFERTY JA AND MARGISON GP. (1993). Depletion of $O^6$-alkylguanine-DNA alkyltransferase correlates with potentiation of temozolomide and CCNU toxicity in human tumour cells. *Br. J. Cancer*, **67**, 1299–1302.

BAKER RM, VAN VOORHIS WC AND SPENCER LA. (1979). HeLa cell variants that differ in sensitivity to monofunctional alkylating agents with independence of cytotoxic and mutagenic responses. *Proc. Natl Acad. Sci. USA*, **76**, 5249–5253.

BODELL WJ, GEROSA M, AIDA T, BERGER MS AND ROSENBLUM ML. (1985). Investigation of resistance to DNA cross-linking agents in 9L cell lines with different sensitivities to chloroethylnitrosoureas. *Cancer Res.*, **45**, 3460–3464.

BRADFORD MM. (1976). A rapid and sensitive method for the quantitation of microgram quantities of protein utilizing the principles of protein-dye binding. *Anal. Biochem.*, **62**, 248–254.

BRANCH P, AQUILINA G, BIGNAMI M AND KARRAN P. (1993). Defective mismatch binding and a mutator phenotype in cells tolerant to DNA damage. *Nature*, **362**, 652–654.

BRANCH P, HAMPSON R AND KARRAN P. (1995). DNA mismatch binding defects, DNA damage tolerance, and mutator phenotypes in human colorectal carcinoma cell lines. *Cancer Res.*, **55**, 2304–2309.

BRENT TP. (1986). Inactivation of purified human $O^6$-alkylguanine-DNA alkyltransferase by alkylating agents or alkylated DNA. *Cancer Res.*, **46**, 2320–2323.

BRENT TP, HOUGHTON PJ AND HOUGHTON JA. (1985). $O^6$-Alkylguanine-DNA alkyltransferase activity correlates with the therapeutic response of human rhabdomyosarcoma xenografts to 1-(2-chloroethyl)-3-(*trans*-4-methyl cyclohexyl)-1-nitrosourea. *Proc. Natl Acad. Sci. USA*, **82**, 2985–2989.

BRENT TP, LESTRUD SO, SMITH DG AND REMACK JS. (1987). Formation of DNA interstrand crosslinks by the novel chloroethylating agent 2-chloroethyl(methylsulfonyl)methane sulfonate: suppression by $O^6$-alkylguanine-DNA alkyltransferase purified from human leukemic lymphoblasts. *Cancer Res.*, **47**, 3384–3387.

CARTER SK AND WASSERMAN TH. (1976). The nitrosoureas: thoughts for the future. *Cancer Treat. Rep.*, **60**, 807–811

CATAPANO CV, BROGGINI M, ERBA E, PONTI M, MARIANI L, CITTI L AND D'INCALCI M. (1987). *In vitro* and *in vivo* methazolastone-induced DNA damage and repair in L1210 leukemia sensitive and resistant to chloroethylnitrosoureas. *Cancer Res.*, **47**, 4884–4889.

CECCOTTI S, DOGLIOTTI E, GANNON J, KARRAN P AND BIGNAMI M. (1993). $O^6$-Methylguanine in DNA inhibits replication *in vitro* by human cell extracts. *Biochemistry*, **32**, 13664–13672.

CESARONE C, BOLOGNESI C AND SANTI L. (1987). Improved microfluorometric DNA determination in biological material using 33258 Hoechst. *Anal. Biochem.*, **102**, 188–197.

CITRON M, DECKER R, CHEN S, SCHNEIDER S, GRAVER M, KLEYNERMAN L, KAHN LB, WHITE A, SCHOENHAUS M AND YAROSH D. (1991). $O^6$-Methylguanine-DNA methyltransferase in human normal and tumour tissue from brain, lung and ovary. *Cancer Res.*, **51**, 4131–4134.

DENNY BJ, WHEELHOUSE RT, STEVENS MFG, TSANG LLH AND SLACK JA. (1994). NMR and molecular modeling investigation of the mechanism of activation of the antitumour drug temozolomide and its interaction with DNA. *Biochemistry*, **33**, 9045–9051.

D'INCALCI M, TAVERNA P, ERBA E, FILIPPESCH S, POTENZA D, MARIANI L, CITTI L AND CATAPANO C. (1991). $O^6$-Methylguanine and temozolomide can reverse the resistance to chloroethylnitrosoureas of a mouse L1210 leukemia. *Anticancer Res.*, **11**, 115–122.

DOLAN ME, CORSICO CD AND PEGG AE. (1985*a*). Exposure of HeLa cells to $O^6$-alkylguanines increases sensitivity to the cytotoxic effects of alkylating agents. *Biochem. Biophys. Res. Commun.*, **132**, 178–185.

DOLAN ME, MORIMOTO K AND PEGG AE. (1985*b*). Reduction of $O^6$-alkylguanine-DNA alkyltransferase activity with $O^6$-alkylguanines. *Cancer Res.*, **45**, 6413–6417.

DOLAN ME, MITCHELL RB, MUMMERT C, MOSCHEL RC AND PEGG AE. (1991). Effect of $O^6$-benzylguanine analogues on sensititivity of human tumour cells to the cytotoxic effects of alkylating agents. *Cancer Res.*, **51**, 3367–3372.

DOLAN ME, PEGG AE, HORA NK AND ERICKSON LC. (1988). Effect of $O^6$-methylguanine on interstrand cross-link formation by chloroethylnitrosoureas and 2-chloroethyl(methylsulfonyl)-methanesulfonate. *Cancer Res.*, **48**, 3603–3606.

DOLAN ME, PEGG AE, MOSCHEL RC AND GRINDLEY GB. (1993). Effect of $O^6$-benzylguanine on the sensitivity of human colon tumour xenografts to 1,3-bis(2-chloroethyl)-1-nitrosourea (BCNU). *Biochem. Pharmacol.*, **46**, 285–290.

DOMORADZKI J, PEGG AE, DOLAN ME, MAHER VM AND MCCORMICK JJ. (1984). Correlation between $O^6$-methylguanine-DNA-methyltransferase activity and resistance of human cells to the cytotoxic and mutagenic effect of *N*-methyl-*N*-nitro-*N*-nitrosoguanidine. *Carcinogenesis*, **5**, 1641–1647.

DRUMMOND JT, LI GM, LONGLEY MJ AND MODRICH P. (1995). Isolation of an hMSH2-p160 heterodimer that restores DNA mismatch repair to tumor cells. *Science*, **268**, 1909–1912.

EDWARDS MS, LEVIN VA AND WILSON CB. (1980). Brain tumour chemotherapy: an evaluation of agents in current use for phase II and III trials. *Cancer Treat. Rep.*, **64**, 1179–1205.

ERICKSON LC, LAURENT G, SHARKEY NA AND KOHN KW. (1980). DNA cross-linking and monoadduct repair in nitrosourea-treated human tumour cells. *Nature*, **288**, 727–729.

FAIRBAIRN LJ, WATSON AJ, RAFFERTY JA, ELDER RH AND MARGISON GP. (1995). O-$^6$-benzylguanine increases the sensitivity of human primary bone marrow cells to the cytotoxic effects of temozolomide. *Exp. Haematol.*, **23**, 112–116.

FELKER GM, FRIEDMAN HS, DOLAN ME, MOSCHEL RC AND SCHOLD C. (1993). Treatment of subcutaneous and intracranial brain tumour xenografts with $O^6$-benzylguanine and 1,3-bis(2-chloroethyl)-1-nitrosourea. *Cancer Chemother. Pharmcol.*, **32**, 471–476.

FISHEL R, LESCOE MK, RAO MRS, COPELAND NG, JENKINS NA, GARBER J, KANE M AND KOLODNER R. (1993). The human mutator gene homolog *MSH2* and its association with hereditary nonpolyposis cancer. *Cell*, **75**, 1027–1038.

FRIEDMAN HS, DOLAN ME, MOSCHEL RC, PEGG AE, FELKER GM, RICH J, BIGNER DD, SCHOLD JR SC. (1992). Enhancement of nitrosourea activity in medulloblastoma and glioblastoma multiforme. *J. Natl Cancer Inst.*, **84**, 1926–1931.

GERSON SL, ZBOROWSKA E, NORTON K, GORDON NH AND WILLSON JKV. (1993). Synergistic efficacy of $O^6$-benzylguanine and 1,3-bis(2-chloroethyl)-1-nitrosourea (BCNU) in a human colon cancer xenograft completely resistant to BCNU alone. *Biochem. Pharmacol.*, **45**, 483–491.

GERSON SL, TREY JE, MILLER K AND BERGER NA. (1986). Comparison of $O^6$-alkylguanine-DNA alkyltransferase activity based on cellular DNA content in human, rat and mouse tissues. *Carcinogenesis*, **7**, 745–749.

GERSON SL, TREY JE AND MILLER K. (1988). Potentiation of nitrosourea cytotoxicity in human leukemic cells by inactivation of $O^6$-alkylguanine-DNA alkyltransferase. *Cancer Res.*, **48**, 1521–1527.

GERSON SL, BERGER NA, ARCE C, PETZOLD SJ AND WILLSON JKV. (1992). Modulation of nitrosourea resistance in human colon cancer by $O^6$-methylguanine. *Biochem. Pharmacol.*, **43**, 1101–1107.

GIBSON NW, HARTLEY JA, BARNES D AND ERICKSON LC. (1986). Combined effects of streptozotocin and mitozolomide against four human cell lines of the $Mer^+$phenotype. *Cancer Res.*, **46**,, 4995–4998.

GOLDMACHER VS, CUZICK RA AND THILLY WG. (1986). Isolation and partial characterization of human cell mutants differing in sensitivity to killing and mutation by methylnitrosourea and *N*-methyl-*N'*-nitro-*N*-nitroguanidine. *J. Biol. Chem.*, **261**, 12462–12471.

GONZAGA PE, POTTER PM, NIU T, YU D, LUDLUM DB, RAFFERTY JA, MARGISON GP AND BRENT TP. (1992). Identification of the cross-link between human $O^6$-methylguanine-DNA methyltransferase and chloroethylnitrosourea-treated DNA. *Cancer Res.*, **52**, 6052–6058.

GOTH-GOLDSTEIN R. (1987). MNNG-induced partial phenotypic reversion of $mer^-$ cells. *Carcinogenesis*, **8**, 1449–1453.

GRIFFIN S AND KARRAN P. (1993). Incision at DNA GT mispairs by extracts of mammalian cells occurs preferentially at cytosine methylation sites and is not targeted by a separate GT binding reaction. *Biochemistry*, **32**, 13032–13039.

GRIFFIN S, BRANCH P, XU Y AND KARRAN P. (1994). DNA mismatch binding and incision at modified guanine bases by extracts of mammalian cells: implications for tolerance to DNA methylation damage. *Biochemistry*, **33**, 4787–4793.

GRILLEY M, WELSH KM, SU SS AND MODRICH P. (1989). Isolation and characterization of the *Escherichia coli mutL* gene product. *J. Biol. Chem.*, **264**. 1000–1004.

HAYWARD IP AND PARSONS PG. (1984). Comparison of virus reactivation, DNA base damage, and cell cycle effects in autologous human melanoma cells resistant to methylating agents. *Cancer Res.*, **44**. 55–58.

HOLMES J, CLARK S AND MODRICH P. (1990). Strand-specific mismatch correction in nuclear extracts of human and *Drosophila melanogaster* cell lines. *Proc. Natl Acad. Sci. USA*, **87**, 5837–5841.

JIANG B, BAWR B, HSIANG Y, SHEN T, POTMESIL M AND SILBER R. (1989). Lack of drug-induced DNA cross-links in chlorambucil-resistant Chinese Hamster Ovary cells. *Cancer Res.*, **49**, 5514–5517.

JIRICNY J, HUGHES M, CORMAN N AND RUDKIN BB. (1988). A human 200-kDa protein binds selectively to DNA fragments containing GT mismatches. *Proc. Natl Acad. Sci. USA*, **85**, 8860–8864.

KARRAN P AND BIGNAMI M. (1992). Self-destruction and tolerance in resistance of mammalian cells to alkylation damage. *Nucleic Acids Res.*, **20**, 2933–2940.

KARRAN P, MACPHERSON P, CECCOTTI S, DOGLIOTTI E, GRIFFIN S AND BIGNAMI M. (1993). $O^6$-Methylguanine residues elicit DNA repair synthesis by human cell extracts. *J. Biol. Chem.*, **268**, 15878–15886.

KAT A, THILLY WG, FANG WH, LONGLEY MJ, LI GM AND MODRICH P. (1993). An alkylation-tolerant, mutator cell line is deficient in strand-specific mismatch repair. *Proc. Natl Acad. Sci. USA*, **90**, 6424–6428.

LEE SM, THATCHER N AND MARGISON GP. (1991). $O^6$-alkylguanine-DNA alkyltransferase depletion and regeneration in human peripheral lymphocytes following Dacarbazine and Fotemustine. *Cancer Res.*, **51**, 619–623.

LEE SM, THATCHER N, CROWTHER D AND MARGISON GP. (1994). Inactivation of $O^6$-alkylguanine-DNA alkyltransferase in human peripheral blood mononuclear cells by temozolomide. *Br. J. Cancer*, **69**, 452–456.

LI G-M AND MODRICH P. (1995). Restoration of mismatch repair to nuclear extracts of H6 colorectal tumour cells by a heterodimer of human MutL homologs. *Proc. Natl Acad. Sci. USA*, **92**, 1950–1954.

LIU L, ALLAY E, DUMENCO LL AND GERSON SL. (1994). Rapid repair of $O^6$-methylguanine-DNA adducts protects transgenic mice from *N*-methylnitrosourea-induced thymic lymphomas. *Cancer Res.*, **54**, 4648–4652.

LOWN JW, MCLAUGHLIN LW AND CHANG YM. (1978). Mechanism of action of 2-haloethylnitrosoureas on DNA and its relation to their antileukemic properties. *Bioorg. Chem.*, **7**, 97–110.

MARATHI UK, KROES RA, DOLAN ME AND ERICKSON LC. (1993). Prolonged depletion of $O^6$-methylguanine DNA methyltransferase activity following exposure to $O^6$-benzylguanine with or without streptozotocin enhances 1,3-bis(2-chloroethyl)-1-nitrosourea sensitivity *in vitro*. *Cancer Res.*, **53**, 4281–4286.

MARATHI UK, DOLAN ME AND ERICKSON LC. (1994). Extended depletion of $O^6$-methylguanine-DNA methyltransferase activity following $O^6$-benzyl-2'-deoxyguanosine or $O^6$-benzylguanine combined with streptozotocin treatment enhances 1,3-bis(2-chloroethyl)-1-nitrosourea cytotoxicity. *Cancer Res.*, **54**, 4371–4375.

MARGISON GP AND O'CONNOR PJ. (1990). Biological consequences of reactions with DNA: Role of specific lesions. Chemical Carcinogenesis and Mutagenesis. In *Handbook of Experimental Chemotherapy*, Grover PL and Phillips DH (eds), pp 547–571, Springer: Heidelberg.

MITCHELL RB, MOSCHEL RC AND DOLAN ME. (1992). Effect of $O^6$-benzylguanine on the sensitivity of human tumour xenografts to 1,3-bis(2-chloroethyl)-1-nitrosourea and on DNA interstrand cross-link formation. *Cancer Res.*, **52**, 1171–1175.

MITCHELL RB AND DOLAN ME. (1993). Effect of temozolomide and dacarbazine on $O^6$-alkylguanine-DNA alkyltransferase activity and sensitivity of human tumour cells and xenografts to 1,3-bis(2-chloroethyl)-1-nitrosourea. *Cancer Chemother. Pharmacol.*, **32**, 59–63.

MITRA G, PAULY GT, KUMAR R, PEI GK, HUGHES SH, MOSCHEL RC AND BARBACID M. (1989). Molecular analysis of $O^6$-substituted guanine-induced mutagenesis of *ras* oncogenes. *Proc. Natl Acad. Sci. USA*, **86**, 8650–8654.

MOERTEL GG. (1973). Therapy of advanced gastrointestinal cancer with the nitrosoureas. *Cancer Chemother. Rep.*, **4**, 27–32.

MONTGOMERY JA, JAMES R, MCCALEB GS AND JOHNSTON TP. (1967). The modes of decomposition of 1,3-bis(2-chloroethyl)-1-nitrosourea and related compounds. *J. Med. Chem.*, **10**, 668–674.

NEWLANDS ES, BLACKLEDGE G, SLACK JA, GODDARD C, BRINDLEY CJ, HOLDEN L AND STEVENS MFG. (1985). Phase I clinical trial of mitozolomide. *Cancer Treat. Rep.*, **69**, 801–805.

NEWLANDS ES, BLACKLEDGE GRP, SLACK JA, RUSTIN GJS, SMITH DB, STUART NSA, QUARTERMAN CP, HOFFMAN R, STEVENS MFG, BRAMPTON MH AND GIBSON AC. (1992). Phase I trial of temozolomide (CCRG 81045: M & B 39831: NSC 362856). *Br. J. Cancer*, **65**, 287–291.

O'REILLY SM, NEWLANDS ES, GLASER MG, BRAMPTON M, RICE EDWARDS JM, ILLINGWORTH RD, RICHARDS PG, KENNARD C, COLQUHOUN IR, LEWIS P AND STEVENS MFG. (1993). Temozolomide: a new oral cytotoxic chemotherapeutic agent with promising activity against primary brain tumours. *Eur. J. Cancer*, **29A** 940–942.

PALOMBO F, HUGHES M, JIRICNY J, TRUONG O AND HSUAN J. (1994). Mismatch repair and cancer. *Nature*, **367**, 417.

PALOMBO F, GALLINARI P, IACCARINO I, LETTIERI T, HUGHES M, D'ARRIGO A, TRUONG O, HSUAN JJ AND JIRICNY J. (1995). GTBP, a 160-kilodalton protein essential for mismatch-binding activity in human cells. *Science*, **268**, 1912–1914.

PANELLA TJ, SMITH DC, SCHOLD SC, ROGERS MP, WINER EP, FINE RL, CRAWFORD J, HERNDON II JE AND TRUMP DL. (1992). Modulation of $O^6$-alkylguanine-DNA alkyltransferase-mediated Carmustine resistance using Steptozotocin: a phase I trial. *Cancer Res.*, **52**, 2456–2459.

PAPADOPOULOS N, NICOLAIDES NC, LIU B, PARSONS R, LENGAUER C, PALOMBO F, D'ARRIGO A, MARKOWITZ S, WILLSON JKV, KINZLER KW, JIRICNY J AND VOGELSTEIN B. (1995). Mutations of GTBP in genetically unstable cells. *Science*, **268**, 1915–1917.

PARSONS R, LI G-M, LONGLEY MJ, FANG W, PAPADOPOULOS N, JEN J, DE LA CHAPELLE A, KINZLER KW, VOLGELSTEIN B AND MODRICH P. (1993). Hypermutability and mismatch repair deficiency in $RER^+$ tumour cells. *Cell*, **75**, 1227–1236.

PEGG AE. (1993). Alkylation and subsequent repair of DNA after exposure to dimethylnitrosamine and related carcinogens. *Rev. Biochem. Toxicol.*, **5**, 83–133.

PEGG AE, SCICCHITANO D AND DOLAN ME. (1984). Comparison of the rates of repair of $O^6$-alkylguanines in DNA by rat liver and bacterial $O^6$-alkylguanine-DNA alkyltransferase. *Cancer Res.*, **44**, 3806–3811.

PIEPER RO, FUTSCHER BW AND ERICKSON LC. (1989). Transcription termination lesions induced by bifunctional alkylating agents *in vitro*. *Carcinogenesis*, **10**, 1307–1314.

PLANT JE AND ROBERTS JJ. (1971). Extension of the pre-DNA synthetic phase of the cell cycle as a consequence of DNA alkylation in Chinese hamster cells: a possible mechanism of DNA repair. *Chemico-Biol. Interact.*, **3**, 343–351.

PLOWMAN J, WAUD WR, KOUTSOUKOS AD, RUBINSTEIN LV, MOORE TD AND GREVER MR. (1994). Preclinical antitumour activity of temozolomide in mice: efficacy against brain tumour xenografts and synergism with 1,3-bis-(2-chloroethyl)-1-nitrosourea. *Cancer Res.*, **54**, 3793–3799.

POTTER DW AND REED DJ. (1983). Involvement of FMN and phenobarbital cytochrome P-450 in stimulating a one electron reductive denitrosation of 1-(2-chloroethyl)-3-(cyclohexyl)-1-nitrosourea catalysed by NADPH-cytochrome P-450 reductase. *J. Biol. Chem.*, **258**, 6906–6911.

REDMOND SMS, JONCOURT F, BUSER K, ZIEMIECKI A, ALTERMATT H, FEY M, MARGISON G AND CERNY T. (1991). Assessment of P-glycoprotein, glutathione-based detoxifying enzymes and $O^6$-alkylguanine-DNA alkyltransferase as potential indicators of constitutive drug resistance in human colorectal tumours. *Cancer Res.*, **51**, 2092–2097.

SARKER A, DOLAN ME, GONZALEZ GG, MARTON LJ, PEGG AE, AND DEEN DF. (1993). The effects of $O^6$-benzylguanine and hypoxia on the cytotoxicity of 1,3-bis(2-chloroethyl)-1-nitrosourea in nitrosourea-resistant SF-763 cells. *Cancer Chemother. Pharmacol.*, **32**, 477–481.

SCUDIERO DA, MEYER SA, CLATTERBUCK BE, MATTERN MR, ZIOLKOWSKI CHJ AND DAY RS III. (1984). Relationship of DNA repair phenotypes of human fibroblast and tumour strains to killing by *N*-methyl-*N*'-nitro-*N*-nitrosoguanidine. *Cancer Res.*, **44**, 961–969.

SEIDENFELD J, BARNES D, BLOCK AL AND ERICKSON LC. (1987). Comparison of DNA intrastrand cross-linking and strand breakage by 1,3-bis(2-chloroethyl)-1-nitrosourea in polyamine-depleted and control human adenocarcinoma cells. *Cancer Res.*, **47**, 4538–4543.

SHIBATA D, PEINADO MA, IONOV Y, MALKHOSYAN S AND PERUCHO M. (1994). Genomic instability in repeated sequences is an early somatic event in colorectal tumorigenesis that persists after transformation. *Nature Genet.*, **6**, 273–281.

SILBER JR, BOBOLA MS, EWERS TG, MURAMOTO M AND BERGER MS. (1992). $O^6$-alkylguanine DNA-alkyltransferase is not a major determinant of sensitivity to 1,3-bis(2-chloroethyl)-1-nitrosourea in four medulloblastoma cell lines. *Oncology Res.*, **4**, 241–248.

SKEHAN P, STORENG R, SCUDIERO D, MONKS A, MCMAHON J, VISTICA D, WARREN JT, BOKESCH H, KENNEY S AND BOYD MR. (1990). New colorimetric assay for anti-cancer drug screening. *J. Natl Cancer Inst.*, **82**, 1107–1118.

SMITH MT, EVANS CG, DOANE-SETZER P, CASTRO VM, TAHIR MK AND MANNERVIK B. (1989). Denitrosation of 1,3-bis(2-chloroethyl)-1-nitrosourea by class $\mu$ glutathione transferases and its role in cellular resistance in rat brain tumour cells. *Cancer Res.*, **49** 2621–2625.

SU SS AND MODRICH P. (1986). *Escherichia coli mutS*-encoded protein binds to mismatched DNA base pairs. *Proc. Natl Acad. Sci. USA*, **83**, 5057–5061.

TANO K, SHIOTA S, COLLIER J, FOOTE RS AND MITRA S. (1990). Isolation and structural characterization of a cDNA clone encoding the human DNA repair protein for $O^6$-alkylguanine. *Proc. Natl Acad. Sci. USA*, **87**, 686–690.

TISDALE MJ. (1987). Antitumour imidazotetrazines-XV. Role of guanine $O^6$ alkylation in the mechanism of cytotoxicity of imidazotetrazinones. *Biochem. Pharmacol.*, **36**, 457–462.

TONG WP, KIRK MC AND LUDLUM DB. (1981). Molecular pharmacology of the haloethyl nitrosoureas: formation of 6-hydroxyethylguanine in DNA treated with BCNU ($N,N^1$-bis[2-chloroethyl]-*N*-nitrosourea). *Biochem. Biophys. Res. Commun.*, **100**, 351–357.

TONG WP, KIRK MC AND LUDLUM DB. (1982). Formation of the cross-link 1-[$N^3$-deoxycytidyl]-2-[$N^1$-deoxyguanosinyl]-ethane, in DNA treated with $N,N^1$-bis(2-chloroethyl)-*N*-nitrosourea (BCNU). *Cancer Res.*, **42**, 3102–3105.

UMAR A, BOYER JC, THOMAS DC, NGUYEN DG, RISINGER JI, BOYD J, IONOV Y, PERUCHO M AND KUNKEL T. (1994). Defective mismatch repair in extract of colorectal and endometrial cell lines exhibiting microsatellite instability. *J. Biol. Chem.*, **269**, 14367–14370.

WALKER MD AND HURWITZ BS. (1970). BCNU (1,3-bis(2-chloroethyl)-1-nitrosourea: NSC 409962) in the treatment of malignant brain tumours. A preliminary report. *Cancer Chemother. Rep.*, **54**, 263–271.

WEINKAM RJ AND LIN HS. (1979). Reactions of BCNU (1,3-bis(2-chloroethyl)-1-nitrosourea) and CCNU (1-[2-chloroethyl]-3-cyclohexyl-1-nitrosourea) in aqueous solution. *J. Med. Chem.*, **22**, 1193–1198.

YANG J, HSIEH F, LEE P AND TSENG HR. (1994). Strand and sequence-specific attenuation of *N*-methyl-*N*'-nitro-*N*-nitrosoguanidine-induced GC to AT transitions by expression of human $O^6$-methylguanine-DNA methyltransferase in Chinese Hamster Ovary cells. *Cancer Res.*, **54**, 3857–3863.

YAROSH DB. (1985). The role of $O^6$-methylguanine-DNA methyltransferase in cell survival, mutagenesis and carcinogenesis. *Mutat. Res.*, **145**, 1–16.

YOUNG RC, DEVITA VT JR, SERPICK AA AND CANELLOS CP. (1971). Treatment of advanced Hodgkin's disease with [1,3-bis(2-chloroethyl)-1-nitrosourea] BCNU., *N. Engl J. Med.*, **285**, 475–479.

ZOUBOULIS C, GARBE C AND ORFANOS CE. (1989). Factors influencing the *in vitro* growth of human melanoma cell lines. *Skin Cancer*, **4**, 63–72.